# Supplementary material for: Mutations in PCYT2 disrupt etherlipid biosynthesis and cause a complex hereditary spastic paraplegia
Source: Brain. 2019 Oct 22;142(11):3382–97. doi: 10.1093/brain/awz291 (PMC6821184; doi:10.1093/brain/awz291)
Supplement: awz291_Supplementary_Data [file awz291_supplementary_data.zip › awz291-Suppl_data/Supplementary_Data4.zip!/Supplementary information 3 - fibroblast lipidomics.zip!/Fibroblast lipidomics/Controle vs Patient2/Summary-boxplots-all.pdf]

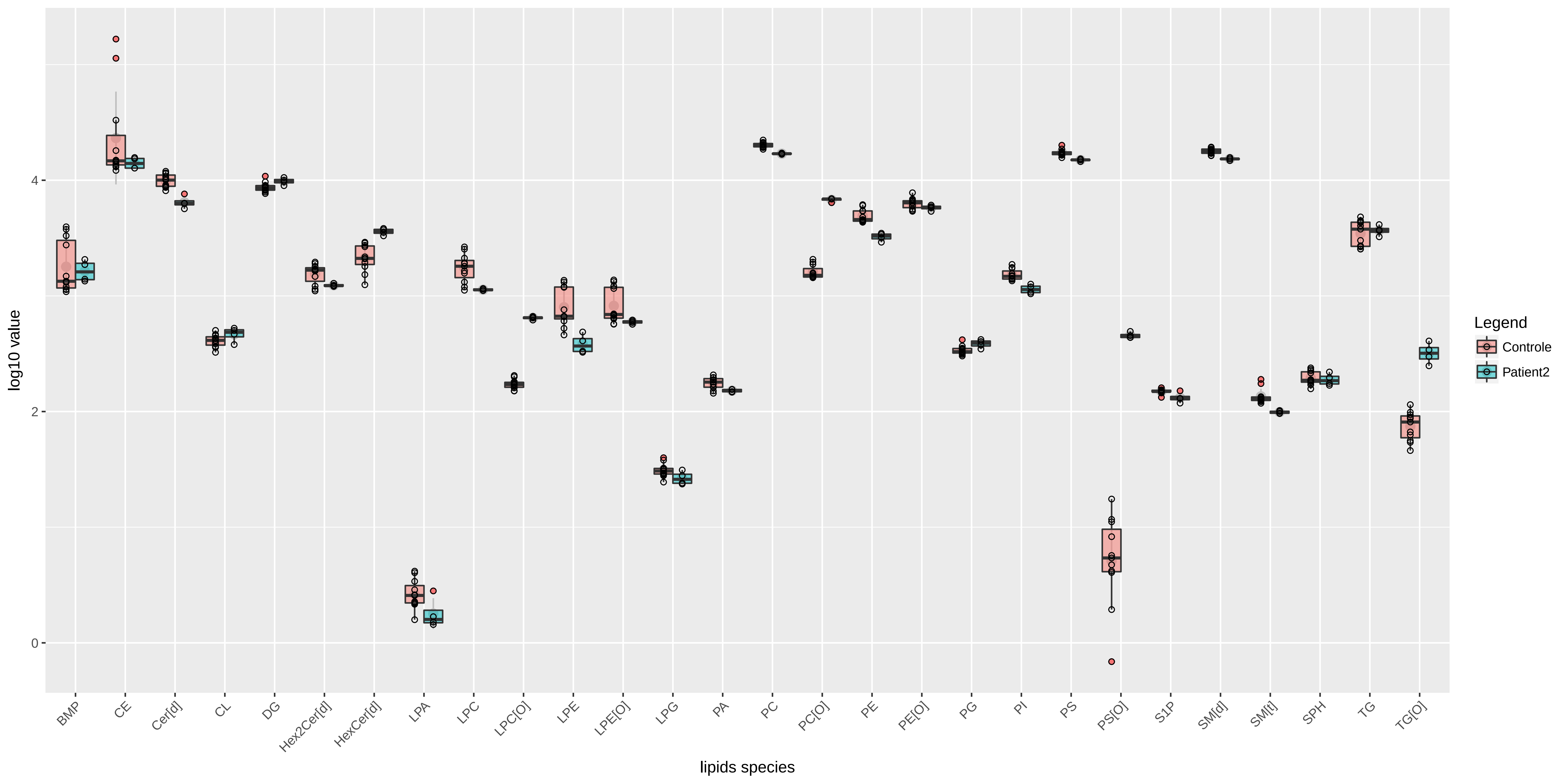

log10 value
0
2
4
BMP
CE
Cer[d]
CL
DG
Hex2Cer[d]
HexCer[d]
LPA
LPC
LPC[O]
LPE
LPE[O]
LPG
PA
PC
PC[O]
PE
PE[O]
PG
PI
PS
PS[O]
S1P
SM[d]
SM[t]
SPH
TG
TG[O]
lipids species
Legend
Controle
Patient2
